# Mapping EBT Store Closures During the COVID-19 Pandemic in a Low-Income, Food-Insecure Community in San Diego

**DOI:** 10.5888/pcd19.210410

**Published:** 2022-06-30

**Authors:** Bryce C. Lowery, Madison R.E. Swayne, Iana Castro, Jessica Embury

**Affiliations:** 1 Gibbs College of Architecture, University of Oklahoma, Norman, Oklahoma; 2 School of Public Affairs, San Diego State University, San Diego, California; 3 Fowler College of Business, San Diego State University, San Diego, California; 4 Department of Geography, San Diego State University, San Diego, California

**Figure:**
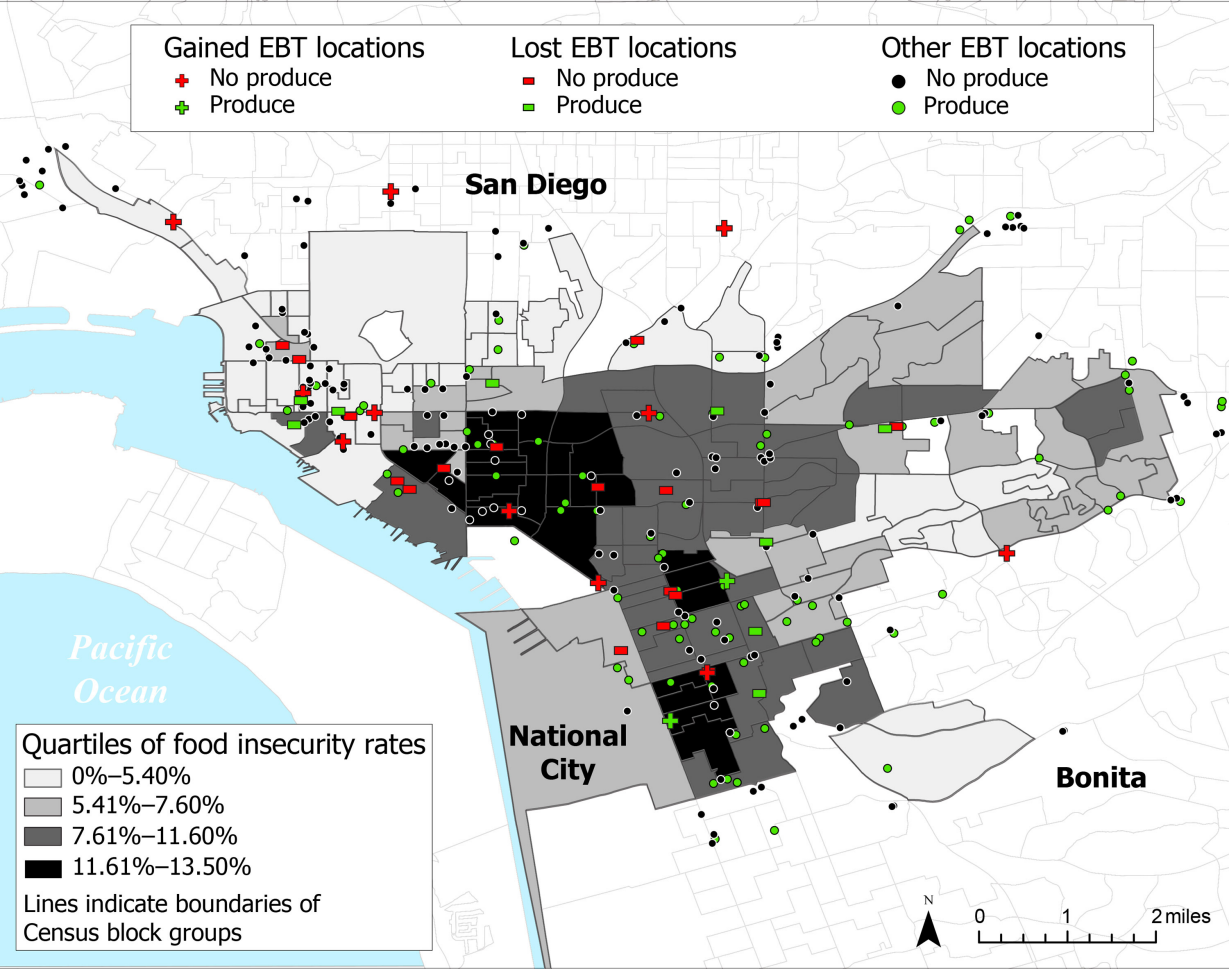
Changes in access to EBT food retailers in Promise Zone communities before (2019) and during (2021) the COVID-19 pandemic, San Diego. Food insecurity rates in 2018 obtained from the California Health Interview Survey AskCHIS Neighborhood Edition (1). EBT store locations obtained from US Department of Agriculture SNAP Retailer Database (2) for July 23, 2019, and July 23, 2021. Abbreviations: EBT, electronic benefits transfer; SNAP, Supplemental Nutrition Assistance Program.

## Background

Access to food retailers that accept electronic benefits transfer (EBT) can help reduce nutritional inequalities among low-income individuals and families experiencing food insecurity. According to the US Department of Agriculture (USDA), nearly all recipients of the Supplemental Nutrition Assistance Program (SNAP) receive benefits via EBT rather than via paper vouchers (3). The transition from physical vouchers to EBT improved enrollment by reducing the stigma associated with paper vouchers and streamlining the process for distribution of benefits (4). Food retailers that accept EBT may be spatially dispersed in ways that make it difficult for low-income residents to access nutritional resources needed to lead a healthy life (5). Proximity to stores that accept EBT supports food security in communities coping with the challenges of poverty.

The COVID-19 pandemic disproportionately affects low-income communities experiencing food insecurity because of 1) the increased risk for infection among people coping with conditions associated with food insecurity and 2) the effects of the pandemic on physical and financial access to sources of nutrition. The pandemic has increased rates of food insecurity (6) by affecting the supply of food and the capacity of individuals to afford food (7,8). People experiencing poverty are at increased risk for COVID-19 (9), and conditions typically associated with food insecurity, such as obesity, diabetes, and cardiovascular disease, are contributors to intensive care admission and in-hospital mortality among patients diagnosed with COVID-19 (10).

The pandemic instigated an economic downturn that shuttered many businesses that provide food, closed schools where children ate, and left many without jobs. During the first month of the pandemic, approximately 30% of US children, particularly those in low-income and racial and ethnic minority groups, experienced household food insecurity (11). In San Diego, the pandemic has had a similar impact: 44% of Black and Hispanic/Latine residents have experienced food insecurity, compared with 25% of the overall population (12). Nationwide, communities responded to these changes; 17% more families applied for SNAP (13) to help mitigate food inaccessibility and unaffordability (14). EBT programs, like Pandemic-EBT, were created to help families purchase food, and evidence suggests that these programs reduced food hardship (6). However, the availability of stores that accept EBT and changes to these stores during the pandemic have not been described in detail.

We expand existing research on food insecurity to explore changes in the availability of stores that accept EBT during the pandemic, including grocery stores and small food retailers like convenience stores and small markets. Our objective was to identify how the availability of stores that accept EBT payments, authorized by SNAP, changed in an area of San Diego County with long-standing patterns of food insecurity.

## Data and Methods

Our study area included 159 low-income census block groups (CBGs) in 4 zip codes in the federally designated San Diego Promise Zone (92101, 92102, 92113, 92114) and 1 zip code from National City (91950), an adjacent neighborhood. Promise Zones are designated by the US Department of Housing and Urban Development (15) as areas that receive special assistance for community revitalization. This study area comprised 15.5 square miles, approximately 279,511 people, and some of San Diego’s most food-insecure zip codes (12,16). We overlaid 2021 land-use data from the San Diego Association of Governments in each CBG. We included CBGs that contain any amount of residential land use, including single family and multifamily.

We downloaded the location of stores that accepted EBT payments from the USDA’s online SNAP Retailer Locator tool (2) on July 23, 2019, and 2 years later, on July 23, 2021. Stores on the EBT list for San Diego County include large-scale supermarkets, small-scale local grocers, specialty markets (eg, bakery, butcher), convenience stores, gas station markets, and liquor stores. We coded each EBT store according to the presence of fresh produce reported previously (17) and locations serviced by BrightSide Produce (www.brightside.sdsu.edu), an initiative designed to support the availability of fruits and vegetables at small markets, convenience stores, and liquor stores that accept EBTs.

We mapped EBT retailer locations and spatially joined them to the 2019 CBG boundaries using ArcGIS Pro version 2.8 (Esri). We used buffer analysis to compute the number of EBT retailers within a ½- mile walking distance (18) of the boundary of each CBG in 2019 and again in 2021 to examine changes after the COVID-19 shutdown. The map of EBT retailers across both periods was overlaid with census tract–level data on food insecurity (proportion of adults aged ≥18 y who are low income and food insecure) obtained from the 2018 California Health Interview Survey AskCHIS Neighborhood Edition online data platform (1); we matched these data to CBG geographies. We used additional CBG-level data from the 2019 American Community Survey (16) to estimate median household income, poverty, education, vehicle ownership, and race and ethnicity. Maps were exported and rendered in Adobe Illustrator 2021. We used Mann–Whitney nonparametric tests to explore differences between each socioeconomic variable retrieved from the US Census for CBGs that lost EBT access and those that gained EBT access. The number of CBGs that gained access to stores that accept EBT with fresh produce was too small to compare (via statistical testing) with the number of CBGs that lost stores that accept EBT with fresh produce, so we reported descriptive statistics only. 

## Highlights

The study area comprised 200 EBT stores on July 23, 2019; by July 23, 2021, twenty-three stores had been removed from San Diego County’s EBT list and 7 stores had been added, resulting in 184 stores (a net loss of 16 [−8.0%] stores). The 23 stores that either closed or stopped accepting EBT were 1 full-service supermarket, 3 bakeries, 3 produce outlets, 1 ice cream shop, 1 pharmacy, 11 convenience stores, 1 gas station, 1 fish market, and a food delivery service. Stores added to the EBT list included 6 convenience stores and 1 pharmacy; the full-service supermarket was not replaced. In 2019, 128 (64.0%) stores offered produce, and in 2021, 121 (65.7%) offered produce (including 2 of 7 new stores). Seven of the 23 closed stores had offered fruits and vegetables. Although fewer stores in 2021 accepted EBT, the percentage of stores that offered produce was similar.

Two-thirds of CBGs (105 of 159; 66.0%) lost access to 1 or more (range, 1–6) EBT stores within ½ mile, and 13 (8.2%) CBGs gained 1 EBT store (Table). Over time, the average number of EBT stores accessible within ½ mile declined by 1.2 stores on average across all CBGs. Mann–Whitney nonparametric tests suggested that the CBGs that lost EBT access, compared with CBGs that gained EBT access, had significantly lower median incomes (*U* = 377.0, *P* = .01), higher poverty rates (*U* = 431.5, *P* = .03), lower high school graduation rates (*U* = 422.0, *P* = .02), a higher proportion of households with no vehicle (*U* = 430.5, *P* = .03), larger Hispanic/Latine populations (*U* = 361.0, *P* = .006), and higher food insecurity rates (*U* = 424.0, *P* = .03). Although we could not use statistical testing, we observed that CBGs that lost EBT stores that carried fresh produce were more varied in socioeconomic composition and experienced lower rates of food security than CBGs that gained fresh produce access.

**Table:** Sociodemographic and Health Characteristics of Census Block Groups (CBGs) in the San Diego Promise Zone and CBGs Inside the Promise Zone That Experienced a Change in EBT (Electronic Benefits Transfer) Access From 2019 to 2021^a^

Determinant	All CBGs in Promise Zone (N = 159)	Gained EBT access (n = 13)	Lost EBT access (n = 105)	Gained EBT access with fresh produce (n = 5)	Lost EBT access with fresh produce (n = 63)
Mean change in EBT access	−1.2	1	−2.0	1	−1.4
Total population	279,511	19,364	186,801	14,310	117,430
% Households <200% of federal poverty level	18.0	13.3	20.7	15.1	17.1
Average median annual household income, $	58,660	74,694	54,202	43,338	59,415
% Adult population (age ≥18 y) with high school diploma	77.1	82.7	52.4	54.4	67.6
% Low-income adults (≥18 y) experiencing food insecurity	7.7	5.1	8.1	10.0	6.8
% Population that does not have a vehicle	12.6	6.4	14.8	5.4	16.0
% Population that is Hispanic/Latine	54.3	38.2	57.0	59.2	48.5
% Population that is Black	11.0	13.9	9.6	8.5	11.5

a Data sources: data on EBT store locations from US Department of Agriculture SNAP Retailer Database (2); data on total population, households <200% federal poverty level; median household income, education, vehicle ownership, and race and ethnicity from the 2019 American Community Survey (16); data on food insecurity from UCLA Center for Health Policy Research (1).

## Action

The loss of EBT stores during the pandemic affected food access to a greater degree among residents in communities experiencing hardships (eg, financial insecurity, lack of vehicle) than in communities experiencing these hardships to a lesser degree. Mapping and monitoring of food insecurity in neighborhoods of concern is crucial as the pandemic continues. Challenges not studied here may affect the number of EBT stores residents can access. As federal income assistance wanes, the demand for food outlets that accept EBT will likely increase. Research on local food landscapes should consider these changing contexts in neighborhoods of long-standing food insecurity. Measures of food retail choice should consider small food retailers, like the ones studied here, along with supermarkets and grocery stores.
